# Chromatin Trapping of Factors Involved in DNA Replication and Repair Underlies Heat-Induced Radio- and Chemosensitization

**DOI:** 10.3390/cells9061423

**Published:** 2020-06-08

**Authors:** Artem V. Luzhin, Bogdan Avanesyan, Artem K. Velichko, Victoria O. Shender, Natalia Ovsyannikova, Georgij P. Arapidi, Polina V. Shnaider, Nadezhda V. Petrova, Igor I. Kireev, Sergey V. Razin, Omar L. Kantidze

**Affiliations:** 1 Institute of Gene Biology Russian Academy of Science, 119334 Moscow, Russia; artyom.luzhin@gmail.com (A.V.L.); avanesyanbogdan@gmail.com (B.A.); velichkoak@gmail.com (A.K.V.); petrova.nadezhda.v@gmail.com (N.V.P.); sergey.v.razin@usa.net (S.V.R.); 2 Center for Precision Genome Editing and Genetic Technologies for Biomedicine, Institute of Gene Biology Russian Academy of Sciences, 119334 Moscow, Russia; 3 Institute for Translational Medicine and Biotechnology, Sechenov First Moscow State Medical University, 119991 Moscow, Russia; 4 Center for Precision Genome Editing and Genetic Technologies for Biomedicine, Federal Research and Clinical Center of Physical-Chemical Medicine of Federal Medical Biological Agency, 119435 Moscow, Russia; shender_vika@mail.ru (V.O.S.); arapidi@gmail.com (G.P.A.); polya.shnaider@yandex.ru (P.V.S.); 5 Shemyakin-Ovchinnikov Institute of Bioorganic Chemistry of the Russian Academy of Sciences, 117997 Moscow, Russia; 6 A.N. Belozersky Institute of Physico-Chemical Biology, Lomonosov Moscow State University, 119992 Moscow, Russia; nat.ovs94@gmail.com (N.O.); kireev@genebee.msu.ru (I.I.K.); 7 Moscow Institute of Physics and Technology (State University), 141701 Moscow, Russia; 8 V.I. Kulakov National Medical Research Center for Obstetrics, Gynecology, and Perinatology, 117997 Moscow, Russia; 9 Department of Biology, Lomonosov Moscow State University, 119992 Moscow, Russia

**Keywords:** hyperthermia, DNA repair, DNA replication, chromatin, PARP

## Abstract

Hyperthermia has been used as an adjuvant treatment for radio- and chemotherapy for decades. In addition to its effects on perfusion and oxygenation of cancer tissues, hyperthermia can enhance the efficacy of DNA-damaging treatments such as radiotherapy and chemotherapy. Although it is believed that the adjuvant effects are based on hyperthermia-induced dysfunction of DNA repair systems, the mechanisms of these dysfunctions remain elusive. Here, we propose that elevated temperatures can induce chromatin trapping (*c*-trapping) of essential factors, particularly those involved in DNA repair, and thus enhance the sensitization of cancer cells to DNA-damaging therapeutics. Using mass spectrometry-based proteomics, we identified proteins that could potentially undergo *c*-trapping in response to hyperthermia. Functional analyses of several identified factors involved in DNA repair demonstrated that *c*-trapping could indeed be a mechanism of hyperthermia-induced transient deficiency of DNA repair systems. Based on our proteomics data, we showed for the first time that hyperthermia could inhibit maturation of Okazaki fragments and activate a corresponding poly(ADP-ribose) polymerase-dependent DNA damage response. Together, our data suggest that chromatin trapping of factors involved in DNA repair and replication contributes to heat-induced radio- and chemosensitization.

## 1. Introduction

Hyperthermia is an anti-cancer treatment that involves tumor heating using an exogenous energy source. Technological advancement has widened the therapeutic window of hyperthermia to 40–45 °C [1,2]. Hyperthermia combined with radio- or chemotherapy improves treatment outcomes which can be explained by multiple factors [3,4]. One mechanism of the sensitizing effect of elevated temperature is the induction of DNA repair dysfunction. Several in vitro, in vivo, and clinical studies have demonstrated that hyperthermia can enhance the beneficial effects of DNA-targeting therapeutic strategies by altering DNA damage response (DDR) pathways [5,6]. However, the molecular mechanisms of this DDR-inhibiting effect of hyperthermia and the complete list of hyperthermia-affected DDR factors remain unknown.

It has recently been proposed that the anticancer cytotoxicity of DNA-binding small molecules can be attributed to their chromatin destabilization properties [7,8]. Curaxins, some anthracyclines, and anthraquinones alter chromatin structure by destabilizing nucleosomes and inducing histone eviction from chromatin. Such chromatin destabilization can promote abnormal trapping of proteins to chromatin (*c*-trapping) and thus lead to the exhaustion of the functional protein pool [8]. This phenomenon was first studied on curaxin-induced *c*-trapping of the FAcilitates Chromatin Transcription (FACT) complex [9,10]. In this study, we questioned whether elevated temperatures could induce *c*-trapping of essential factors, particularly those involved in DNA repair, and thus stimulate the sensitization of cancer cells to DNA-damaging therapeutics.

Using mass spectrometry (MS)-based proteomics, we identified an extensive list of proteins that could potentially undergo *c*-trapping in response to hyperthermia. Functional analyses of several identified factors involved in DNA repair demonstrated that *c*-trapping could indeed be the mechanism of hyperthermia-induced transient inactivation of DDR pathways. In addition, we found new evidence for the devastating effect of hyperthermia on DNA replication. Based on the proteomics data obtained, we hypothesized and verified that hyperthermia inhibits maturation of Okazaki fragments and provokes corresponding poly(ADP-ribose) polymerase (PARP)-dependent DDR. Together, our data suggest that chromatin trapping of factors involved in DNA repair and replication underlies heat-induced radio- and chemosensitization.

## 2. Materials and Methods

### 2.1. Antibodies

The primary antibodies used for immunofluorescence or Western blot hybridization were SUPT16H (mouse, Abnova, Taipei, Taiwan), Ku80 (mouse, Abcam, Cambridge, UK), MDC1 (mouse, Abcam), Mre11 (rabbit, Novus Biologicals, Centennial, CO, USA), 53BP1 (rabbit, Santa Cruz Biotechnology, Dallas, TX, USA), XRCC1 (rabbit, Abcam), TopBP1 (mouse, Santa Cruz Biotechnology), Lamin B1 (mouse, Abcam), histone H3 (rabbit, Abcam), PAR (rabbit, Trevigen, Gaithersburg, MD, USA). The secondary antibodies conjugated to either Alexa Fluor 488 or Alexa Fluor 594 were purchased from Invitrogen (Karlsbad, CA, USA); the horseradish peroxidase-conjugated anti-mouse and anti-rabbit IgG were purchased from GE Healthcare (Chicago, IL, USA).

### 2.2. Cell Culture, Drug Treatments, and Hyperthermia

Human HeLa or HEK293 cells were obtained from ATCC. The cells were cultured in Dulbecco’s Modified Eagle’s Medium (DMEM; PanEco, Moscow, Russia) supplemented with 10% fetal bovine serum (FBS; GE Healthcare) at 37 °C in a humidified CO2 incubator. For hyperthermia, cells were immersed in a precision-controlled water bath at 42–45 °C (±0.05 °C) for 30 min. To induce double-stranded DNA breaks (DSBs), cells were treated with 20 µg/mL etoposide (Sigma-Aldrich, St. Louis, MO, USA) for 1 h; to induce single-stranded DNA breaks (SSBs), cells were treated with 200 µM peroxide hydrogen (Sigma-Aldrich) for 1 h; to induce replication stress, cells were treated with 10 mM hydroxyurea (Sigma-Aldrich) or with 10 µM aphidicolin (Sigma-Aldrich) for 1 h; for inhibition of lagging strand synthesis, cells were treated with 2 mM emetine (Sigma-Aldrich) for 1 h; for *c*-trapping induction, cells were treated with 10 µM curaxin CBL0137 (Selleckchem, Houston, TX, USA) for 1 h. To label active replication sites, cells were incubated with 10 μM 5-ethynyl-2’-deoxyuridine (EdU; Jena Biosciences, Jena, Germany) for 20 min at 37 °C. Incorporated EdU were visualized using a Click−iT EdU Imaging Kit (Invitrogen) according to the manufacturer’s instructions.

### 2.3. Chromatin Enriching Salt Separation and Immunoblotting

HeLa cells were incubated in a lysis buffer (LB; 10 mM Hepes-NaOH (pH 7.5), 1.5 mM MgCl_2_, 0.5 mM EDTA, 10 mM KCl, 0.5% NP40, phosphatase and protease inhibitors). Cells were incubated at 4 °C for 10 min and collected by centrifugation at 1000× *g* for 5 min. Cells were then incubated in an LB containing 100 mM NaCl. After incubation at 4 °C for 10 min, the first soluble fraction (“0.1” fraction) was separated by centrifugation at 10,000× *g* for 10 min. Cells were then incubated in an LB containing 400 mM NaCl. After incubation at 4 °C for 1 h, the second soluble fraction (“0.4” fraction) was separated from the chromatin fraction by centrifugation at 8000× *g* for 10 min. The chromatin pellet (“insoluble” fraction) was then sonicated in an LB at 1/2 amplitude for 30 s with a VirSonic 100 ultrasonic cell disrupter.

Aliquots of each sample were separated by sodium dodecylsulphate-polyacrylamide gel electrophoresis (SDS-PAGE) and blotted onto polyvinylidenedifluoride (PVDF) membranes. The membranes were blocked for 1 h in 2% ECL Advance blocking reagent (GE Healthcare) or 2% bovine serum albumin (BSA) (Sigma-Aldrich) in PBS containing 0.1% Tween 20 (PBS-T) followed by incubation overnight at 4 °C with a primary antibody diluted in PBS-T containing 2% blocking reagent or 2% BSA. After three washes with PBS-T, the membranes were incubated for 1 h with the secondary antibodies (horseradish peroxidase-conjugated anti-rabbit or anti-mouse IgG) in PBS-T containing 2% blocking agent or 2% BSA. The immunoblots were visualized using a Pierce ECL plus Western blotting substrate. Images of the full-length blots are presented in Figures S1 and S2.

### 2.4. Preparative SDS-PAGE and In-Gel Trypsin Digestion

Protein concentrations were determined using BCA Protein Assay Kit (Thermo Fisher Scientific, Waltham, MA, USA) according to the manufacturer’s standard protocol (bovine serum albumin was used as the standard). Equal amounts of biological samples (250 μg each) were separated via 9% (*w*/*v*) SDS-PAGE (20 × 20 cm) to prefractionate the proteins. After electrophoresis, the gel was divided into three fractions, each fraction was subjected to in-gel protein digestion, and extracted peptide samples were analyzed via LC-MS/MS for protein identification. Gel fractions were cut into small (1 × 1 mm) pieces and transferred into sample tubes. Protein disulfide bonds were reduced with 10 mM DTT (in 100 mM ammonium bicarbonate buffer) at 50 °C for 30 min and afterward alkylated with 55 mM iodoacetamide (in 100 mM ammonium bicarbonate buffer) at room temperature for 20 min in the dark. After alkylation, the gel samples were destained with 50% acetonitrile (CAN) (in 50 mM ammonium bicarbonate buffer) and dehydrated by the addition of 100% ACN. After removal of the 100% ACN, the samples were subjected to in-gel trypsin digestion. The digestion buffer contained 13 ng/µL trypsin (in 50 mM ammonium bicarbonate buffer). The trypsin digestion proceeded overnight at 37 °C. The resulting tryptic peptides were extracted from the gel via the addition of two volumes of 0.5% trifluoroacetic acid (TFA) to the samples (incubation for 1 h) and then two volumes of 50% ACN (incubation for 1 h). Finally, the extracted peptides were dried in vacuum and redissolved in 3% ACN with 0.1% TFA solution prior to LC-MS/MS analysis.

### 2.5. LC-MS/MS Analysis

LC-MS/MS analysis was performed using the Q Exactive HF benchtop Orbitrap mass spectrometer (Thermo Fisher Scientific) which was coupled to the Ultimate 3000 Nano LC System (Thermo Fisher Scientific) via a nanoelectrospray source (Thermo Fisher Scientific). The HPLC system was configured in a trap-elute mode. Approximately 1 µg of tryptic peptides were loaded on an Acclaim PepMap 100 (100 µm × 2 cm) trap column and separated on an Acclaim PepMap 100 (75 µm × 50 cm) column (both from Thermo Fisher Scientific). Peptides were loaded in solvent A (0.2% (*v*/*v*) formic acid) and eluted at a flow rate of 350 nL/min with a following multistep linear gradient of solvent B (0.1% (*v*/*v*) formic acid, 80% (*v*/*v*) acetonitrile): 4–6% B for 5 min; 6–28% B for 91 min; 28–45% B for 20 min; 45–99% B for 4 min; 99% B for 7 min; 99–4% B for 1 min. After each gradient, the column was washed with 96% buffer B for 9 min. Column temperature was kept at 40 °C. Peptides were analyzed on a mass-spectrometer, with one full scan (350–1400 *m*/*z*, R = 60,000 at 200 *m*/*z*) at a target of 3 × 10^6^ ions and max ion fill time 30 ms, followed by up to 15 data-dependent MS/MS scans with higher-energy collisional dissociation (HCD) (target 1 × 10^5^ ions, max ion fill time 50 ms, isolation window 1.2 *m*/*z*, normalized collision energy (NCE) 28%, underfill ratio 2%), detected in the Orbitrap (R = 15,000 at fixed first mass 100 *m*/*z*). Other settings: charge exclusion—unassigned, 1, >6; peptide match—preferred; exclude isotopes—on; dynamic exclusion 30 s was enabled.

### 2.6. LC-MS/MS Data Analysis

Raw LC-MS/MS data from Q Exactive HF mass-spectrometer were converted to .mgf peaklists with MSConvert (ProteoWizard Software Foundation). For this procedure, we used the following parameters: “--mgf-filter peakPicking true”. For thorough protein identification, the generated peak lists were searched with MASCOT (version 2.5.1) and X! Tandem (ALANINE, 2017.02.01) search engines against UniProt Human protein knowledgebase with the concatenated reverse decoy dataset. The precursor and fragment mass tolerance were set at 20 ppm and 0.04 Da, respectively. Database-searching parameters included the following: tryptic digestion with one possible missed cleavage, static modification for carbamidomethyl (C), and dynamic/flexible modifications for oxidation (M). For X! Tandem we also selected parameters that allowed a quick check for protein N-terminal residue acetylation, peptide N-terminal glutamine ammonia loss, or peptide N-terminal glutamic acid water loss. Result files were submitted to Scaffold 4 software (version 4.0.7) for validation and meta-analysis. We used the local false discovery rate-scoring algorithm with standard experiment-wide protein grouping. For the evaluation of peptide and protein hits, a false discovery rate of 5% was selected for both. False positive identifications were based on reverse database analysis. We also set protein annotation preferences in Scaffold to highlight Swiss-Prot accessions among others in protein groups. To highlight protein functional groups in the identified lists of proteins the Reactome pathway enrichment analysis was applied [11]. The FDR value was obtained from over-representation analysis and was calculated using the Benjamini–Hochberg approach [11].

### 2.7. Immunofluorescence Microscopy (Including Super-Resolution Microscopy)

For immunostaining, cells were grown on microscope slides. All samples were fixed in CSK buffer (10 mM PIPES, pH 7.0, 100 mM NaCl, 1.5 mM MgCl_2_, 300 mM sucrose) supplemented with 1% paraformaldehyde (PFA) and 2.5% Triton X-100 for 15 min at room temperature. Cells were washed in PBS and then were incubated with antibodies in PBS supplemented with 1% BSA and 0.05% Tween 20 for 1 h at room temperature or overnight at 4 °C. Then the cells were washed three times (5 min each time) with PBS. The primary antibodies bound to antigens were visualized using Alexa Fluor 488-conjugated secondary antibodies. The DNA was counterstained with the fluorescent dye 4,6-diamino-2-phenylindole (DAPI) for 10 min at room temperature. The samples were mounted using Dako fluorescent mounting medium (Life Technologies). The immunostained samples were analyzed using a Zeiss AxioScope A.1 fluorescence microscope (objectives: Zeiss N-Achroplan 40 ×/0.65 and EC Plan-Neofluar 100 ×/1.3 oil; camera: Zeiss AxioCam MRm; acquisition software: Zeiss AxioVision Rel. 4.8.2; Jena, Germany). The images were processed using ImageJ software (version 1.44). The images were analyzed using CellProfiler software (version 3.1.5).

Samples for structured illumination microscopy (SIM) were mounted in Dako fluorescent mounting medium (Life Technologies, Karlsbad, CA, USA) and examined using a Nikon N-SIM microscope (100×/1.49 NA oil immersion objective, 488 and 561 nm diode laser excitation). Image stacks (z-steps of 120 nm) were acquired with an EMCCD camera (Andor iXon 897, effective pixel size 60 nm). Exposure conditions were adjusted to get a typical yield of about 5000 max counts (16-bit raw image) while keeping bleaching minimal. Image acquisition, SIM image reconstruction, and data alignment were performed using NIS-Elements (Nikon, Tokyo, Japan).

### 2.8. Live-Cell Imaging and FRAP Analysis

Two days prior imaging cells were transiently transfected with plasmid encoding eGFP-53BP1 which was a gift from Daniel Durocher (Addgene plasmid #60813; http://n2t.net/addgene:60813; RRID:Addgene_60813, Watertown, MA, USA). Live cell imaging was performed at 37 °C and 5% CO_2_ in a humidified stage top incubator (Tokai Hit, Shizuoka, Japan). Nikon Ti-E microscope with 60× Plan Apo oil objective (NA 1.4) and Nikon C2plus camera was used to collect multipoint time-lapse live cell images for 1 h with a temporal resolution of 5 min/frame. Imaging conditions were adjusted to minimize phototoxicity. 

FRAP experiments were performed on the Nikon Ti-E microscope with 60× Plan Apo oil objective (NA 1.4) and Nikon C2plus camera at 37 °C in a humidified incubator. For bleaching and imaging of eGFP-53PB1, a 488-nm laser was used. After bleaching cells were imaged every 5 s for 5 min. Resulted images were analyzed using ImageJ software (version 1.44).

### 2.9. In Situ Nick Translation

Cells were fixed and permeabilized in CSK buffer supplemented with 1% PFA and 2.5% Triton X-100 for 15 min at room temperature. The cells were incubated overnight at 37 °C with a 70 μL nick translation reaction buffer containing 50 mM Tris-HCI (pH 7.5), 10 mM MgCl2, 1 mM DTT, 1 U of *Escherichia coli* DNA polymerase I (New England Biolabs, Ipswich, MA, USA), 10 μM each of dATP, dGTP, dCTP, and dTTP (Sileks, Moscow, Russia), and 3 μM fluorescein-labeled dUTP. The reaction was terminated by incubation the slides in PBS; the slides were then used for immunostaining. For positive control, fixed cells were treated with RNase-free DNase I (1 U/mL; New England Biolabs) for 30 min at room temperature in PBS.

## 3. Results

### 3.1. Hyperthermia Induces C-Trapping

**Figure 1:**
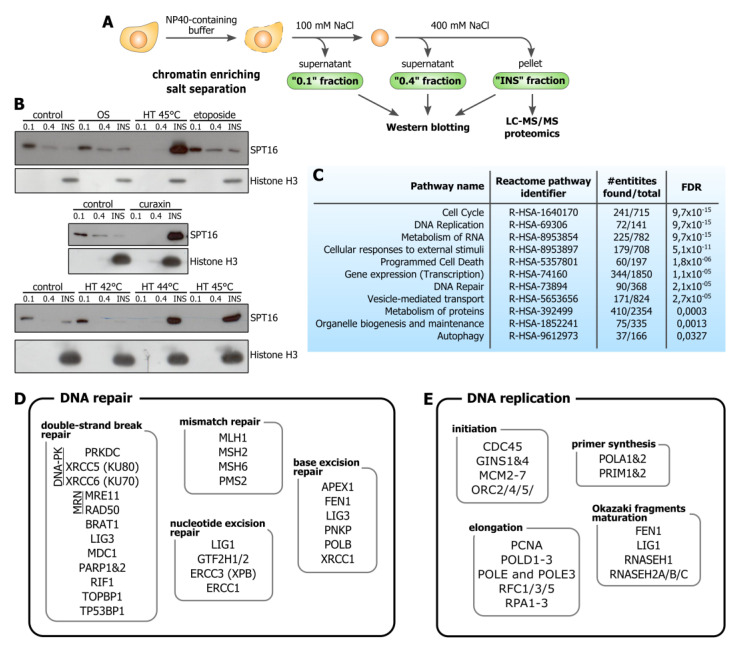
Identification of proteins that can be trapped to chromatin by hyperthermia. (**A**) Schematic representing chromatin enriching salt separation used in this study. Protein fractions obtained using this approach (green boxes) were analyzed by Western blotting or LC-MS/MS-based proteomics. (**B**) Western blot analysis of SPT16 (FAcilitates Chromatin Transcription (FACT) subunit) and histone H3 in different chromatin fractions (see (**A**)) prepared from control HeLa cells and HeLa cells treated either with hypoosmotic stress (OS; 150 mOsmol, 60 min), hyperthermia (HT; 42, 44, or 45 °C, 30 min), etoposide (20 µg/mL, 60 min) or curaxin CBL0137 (10 µM, 60 min). (**C**) Significantly enriched Reactome top-level (parent-level) pathway terms in the list of proteins undergoing *c*-trapping in response to hyperthermia. FDR, false discovery rate. For additional details, see Supplementary Materials Table S2. (**D**,**E**) DNA repair (**D**) and DNA replication (**E**) proteins identified as *c*-trapped in hyperthermia are grouped according to their role in specific pathways.

We first sought to analyze whether mild hyperthermia can induce *c*-trapping comparable to curaxins and other DNA-binding small-molecule drugs [7]. We applied chromatin-enriching salt separation [12] (Figure 1A) to study the distribution of SPT16, a FACT complex subunit, among protein fractions differentially associated with chromatin. In contrast to other treatments (hypo-osmotic stress, etoposide treatment), short-term hyperthermia (44–45 °C, 30 min) caused a rapid and complete redistribution of SPT16 to the insoluble chromatin fraction (Figure 1B). These results show that hyperthermia can potentially induce *c*-trapping in living human cells. We identified as comprehensive of a list as possible of all proteins that undergo *c*-trapping in response to short-term hyperthermia. 

The insoluble chromatin fractions (Figure 1A) obtained from intact HeLa cells and hyperthermia-treated cells (45 °C, 30 min) were analyzed by MS-based proteomics. Differential enrichment analysis of the proteomics data showed that more than 1500 proteins were enriched in, and fewer than 100 proteins were depleted from, the insoluble chromatin fraction upon hyperthermia (Dataset S1). Most of the factors depleted from the insoluble fraction were in some way related to ribosome biogenesis (Table S1). This reflects the hyperthermia-mediated silencing of RNA polymerase I-dependent transcription and nucleolar segregation [13]. We used the Reactome pathway enrichment analysis [11] to highlight protein functional groups associated with the list of factors overrepresented in the insoluble chromatin fraction upon hyperthermia (Figure 1C and Table S2). The observed enrichment for some groups—such as ‘Cell cycle’, ‘Cellular responses to external stimuli’, ‘Programmed cell death’, ‘Autophagy’, and others—can be explained by the general upregulation of the corresponding genes in the course of a cell stress response. However, it seems most important that this list of proteins was enriched with factors involved in DNA replication and repair (Figure 1C).

### 3.2. Hyperthermia-Induced C-Trapping Causes DNA Repair Deficiency

Functional annotation of DNA repair proteins that underwent *c*-trapping in response to hyperthermia demonstrated that almost all major repair pathways were affected (Figure 1D). This includes DNA double-strand break (DSB) repair, base and nucleotide excision repair systems (BER and NER), and mismatch repair (MMR) (Figure 1D and Table S2). Three pathways of DSB repair were affected: classic and alternative nonhomologous end-joining systems (c-NHEJ and alt-NHEJ) and homologous recombination. DNA-dependent protein kinase (DNA-PK) and MRN complex (Mre11-Rad50-Nbs1) subunits, which have been reported to be altered in some way by hyperthermia [14,15,16,17], were *c*-trapped by hyperthermia among other proteins. Within the MRN complex, we only observed the Mre11 and Rad50 subunits, which may interact with the DNA/chromatin, but not the Nbs1 subunit, which did not bind DNA [18]. This also suggests that proteins are trapped to chromatin.

Our data generally corroborate the idea that hyperthermia slightly alters NER [5] because only the TFIIH subunits, ERCC1 and DNA ligase I were *c*-trapped upon hyperthermia. At the same time, most of the crucial BER and MMR factors underwent hyperthermia-induced *c*-trapping, which aligns perfectly with the fact that these repair systems were among the most altered by hyperthermia [4,19]. It is also noteworthy that almost all DNA repair factors that have been reported to be functionally inactivated by hyperthermia (e.g., DNA-PK, MRN, DNA polymerase β, XRCC1) were found among the identified *c*-trapped proteins. This provides evidence that *c*-trapping can indeed be a mechanism of hyperthermia-induced exhaustion of DNA repair factors and subsequent radio and chemosensitization of cells.

**Figure 2:**
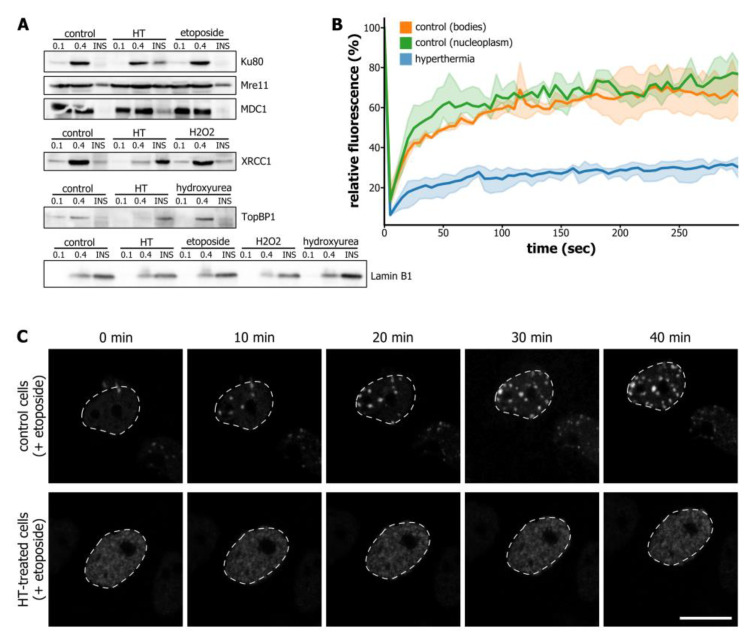
Hyperthermia-induced *c*-trapping can be a mechanism of DNA repair deficiency. (**A**) Western blot analysis of Ku80, Mre11, MDC1, XRCC1, and TopBP1 in different chromatin fractions (see Figure 1A) prepared from control HeLa cells and HeLa cells treated either with hyperthermia (45 °C, 30 min) or specific DNA damage-inducing drug (etoposide, 20 µg/mL, 60 min; hydrogen peroxide (H_2_O_2_), 200 µM, 60 min; hydroxyurea, 10 mM, 60 min). Lamin B1 is used as a loading control. (**B**) Kinetics of 53BP1-GFP studied by fluorescence recovery after photobleaching (FRAP) in control HeLa cells and HeLa cells exposed to hyperthermia (45 °C, 30 min). In control cells, the kinetics of 53BP1-GFP was analyzed both in 53BP1 nuclear bodies and in the nucleoplasm. The shaded parts of the lines represent s.d., *n* = 9. (**C**) Transiently transfected with 53BP1-GFP HeLa cells were mock-treated or treated with hyperthermia (45 °C, 30 min) and then incubated with DNA double-strand break (DSB)-inducing drug etoposide (20 µg/mL, 60 min). Time-lapse imaging of 53BP1-GFP was performed. A representative image is shown. Scale bar: 10 µm.

One might suggest that *c*-trapping of DNA repair factors reflects their functional association with DNA/chromatin in the course of the DDR. To ensure that *c*-trapping is not a part of the working cycles of DNA repair factors but primarily represents their non-functional sequestration, we tested whether these proteins are trapped to chromatin in cells treated with various DNA-damaging agents. This was performed using chromatin-enriching salt separation combined with Western blotting (Figure 1A). We studied the distribution of factors representing the DSB repair system (Ku70, Mre11, MDC1), NER (XRCC1), and DNA replication stress (TopBP1) in different chromatin fractions. The drugs were chosen so that they would induce DSBs (etoposide), SSBs (hydrogen peroxide), or DNA replication stress (hydroxyurea). In accordance with the proteomics analysis, short-term hyperthermia induced the rapid redistribution of the studied proteins to the insoluble chromatin fraction (Figure 2A). At the same time, induction of relevant DNA lesions did not alter protein distribution between chromatin fractions as compared to the control cells (Figure 2A). These results show that DNA repair proteins do not redistribute to the insoluble chromatin fraction during their functional activity. It is thus likely that such redistribution represents protein sequestration that can lead to exhaustion of active protein pools.

To confirm that hyperthermia induces protein trapping to chromatin, we utilized the fluorescence recovery after photobleaching (FRAP) technique. We assumed that *c*-trapping would decrease the mobility of proteins. Using FRAP, we analyzed the dynamics of the 53BP1-GFP protein in control HeLa cells and in cells exposed to short-term hyperthermia (45 °C, 30 min). In control cells, we analyzed the dynamics of 53BP1-GFP in so-called 53BP1-containing bodies [20] and the nucleoplasm. In both cases, the dynamics were within the seconds range (Figure 2B), which is in agreement with the published data [21]. The mobility of 53BP1-GFP in the nucleoplasm decreased substantially in the cells exposed to hyperthermia (Figure 2B). Moreover, following photobleaching, the fluorescence of 53BP1-GFP did not completely recover for more than 10 min. These results clearly show that hyperthermia not only decreases the dynamics of 53BP1-GFP but also completely exhausts the pool of the free high-mobility fraction of 53BP1-GFP.

Finally, we verified whether hyperthermia affects the functionality of the *c*-trapped DNA repair proteins. Again, we used 53BP1-GFP to track DNA repair foci formation in response to the DSB-inducing drug etoposide in HeLa cells exposed or not exposed to hyperthermia. In control cells, etoposide rapidly induced the appearance of large 53BP1-GFP foci within the first 10 min of the treatment (Figure 2C). In HeLa cells that had been exposed to hyperthermia (45 °C, 30 min) and then treated with etoposide, 53BP1-GFP could not form DNA repair foci, even during 60–90 min of tracking (Figure 2C). Thus, using the example of 53BP1, we demonstrated that hyperthermia could indeed cause a DNA repair deficiency by inducing the *c*-trapping of DNA repair proteins.

### 3.3. Hyperthermia Inhibits Maturation of Okazaki Fragments and Provokes a Corresponding PARP-Dependent DNA Damage Response

**Figure 3:**
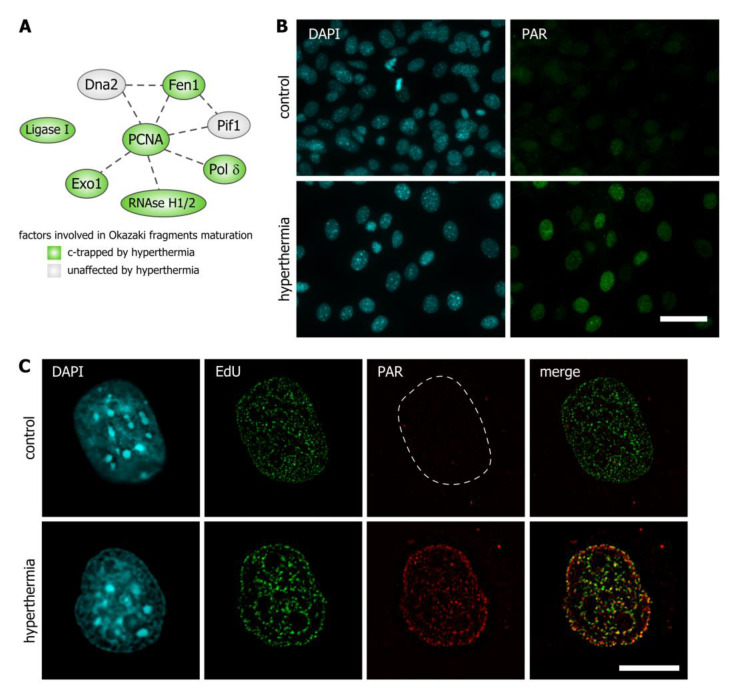
Hyperthermia induces DNA replication-associated poly(ADP-ribosyl)ation (PARylation). (**A**) The network of proteins involved in the maturation of Okazaki fragments in human cells. Proteins identified as *c*-trapped in response to hyperthermia are shown in green ellipses. (**B**) HEK293 cells were subjected to hyperthermia (45 °C, 30 min) and stained with antibodies against PAR. Control represents HEK293 cells that were not exposed to hyperthermia. The DNA was stained with 4,6-diamino-2-phenylindole (DAPI) (blue). Epifluorescence microscopy analysis was performed. Scale bar: 40 µm. (**C**) HEK293 cells were pulse-labeled with 5-ethynyl-2’-deoxyuridine (EdU) (10 µM, 30 min), subjected to hyperthermia (45 °C, 30 min) and stained with antibodies against PAR. Control represents untreated HEK293 cells. EdU was revealed by Click Chemistry; the DNA was stained with DAPI. Structured illumination microscopy (SIM) analysis was performed. Scale bar: 5 µm.

Many components of DNA replication machinery were present in a group of proteins that had undergone *c*-trapping in response to hyperthermia. All stages of the DNA replication process were affected by hyperthermia, including initiation and primer synthesis, elongation, and maturation of Okazaki fragments (Figure 1D). It is well-established that short-term hyperthermia can inhibit DNA replication [22,23]. The observed *c*-trapping of factors involved in DNA replication can explain this phenomenon. Interestingly, hyperthermia led to the *c*-trapping of DNA ligase I, flap structure-specific endonuclease 1 (FEN1), ribonucleases H1 and H2, PCNA, and DNA polymerase δ, each indispensable for processing and ligation of Okazaki fragments (Figure 3A and Dataset S1). We hypothesized that the prevention of Okazaki fragment maturation would result in SSB stabilization on the lagging DNA strand in the vicinity of DNA replication forks. This, in turn, would lead to the activation of the PARP-dependent DDR. Indeed, immunostaining of HEK293 cells with an antibody against poly(ADP-ribose) (PAR) demonstrated that short-term hyperthermia (45 °C, 30 min) induced substantial nuclear poly(ADP-ribosyl)ation (PARylation) in S-phase cells (Figure 3A,B). We applied super-resolution microscopy (SIM) to investigate whether hyperthermia-induced PARylation occurred at sites of DNA replication (Figure 3C). Almost complete co-localization of PAR and EdU-labelled replication foci was observed in HEK293 cells exposed to short-term hyperthermia (Figure 3C).

**Figure 4:**
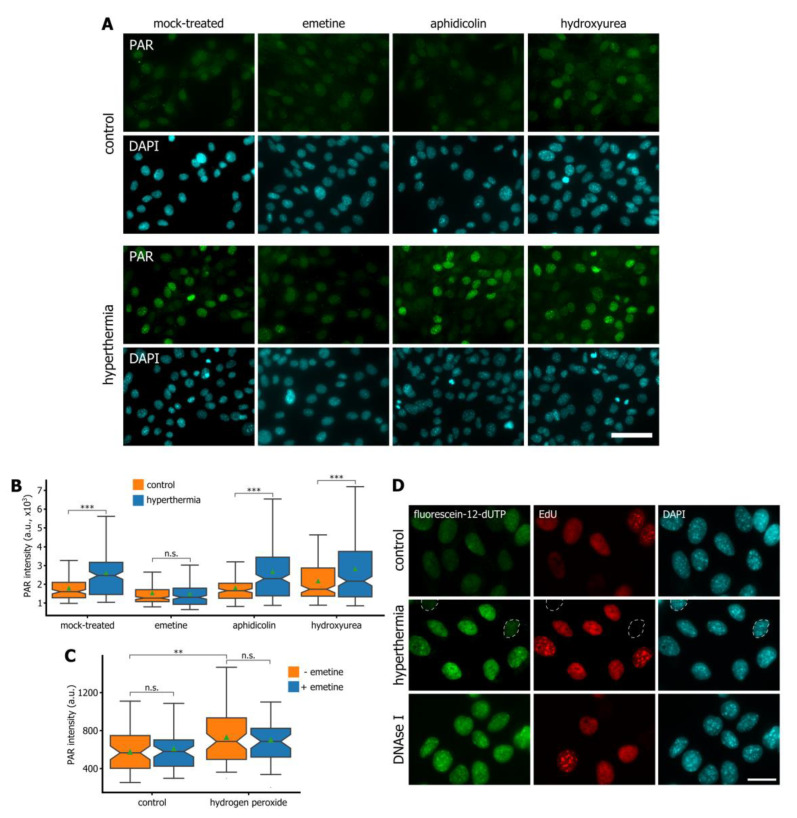
Suppression of Okazaki fragment formation with emetine prevents hyperthermia-induced PARylation. (**A**) HEK293 cells were mock-treated or treated with either emetine (2 mM, 1 h), aphidicolin (10 µM, 1 h), or hydroxyurea (10 mM, 1 h) and then exposed or not exposed to hyperthermia (45 °C, 30 min). The cells were immunostained for PAR. The DNA was stained with DAPI. Scale bar: 50 µm. (**B**) Quantification of PAR in HEK293 cells treated as in (**A**). Box plots show the PAR fluorescence intensities. The horizontal lines represent the median values; the triangles represent the average values. ***p < 0.0001, n.s.—not significant (two-tailed *t*-test, *n* > 100). (**C**) HEK293 cells were mock-treated or treated with emetine (2 mM, 1 h) and then treated or not with hydrogen peroxide (200 µM, 1 h). The cells were stained with antibodies against PAR. Box plots show the PAR fluorescence intensities. The horizontal lines represent the median values; the triangles represent the average values. **p < 0.001, n.s.—not significant (two-tailed *t*-test, *n* > 50). (**D**) HEK293 cells were pulse-labeled with EdU (10 µM, 30 min), exposed to hyperthermia (45 °C, 30 min), fixed, permeabilized, and subjected to a fluorescein-labeled nucleotide analog incorporation assay using *Escherichia coli* DNA polymerase I. Control represents the cells that were not exposed to hyperthermia. Nuclei of the EdU-negative cells were marked by dashed circles on the middle panel to show that the hyperthermia induced SSBs only in S-phase cells. HEK293 cells that were fixed and then treated with DNase I (1 U/mL, 30 min) were used as an additional, positive, control. EdU was revealed by Click Chemistry; the DNA was stained with DAPI. Scale bar: 20 µm.

The results obtained suggest that hyperthermia-induced PARylation could indeed depend on the perturbation of Okazaki fragment maturation. To confirm that Okazaki fragments were the source of hyperthermia-induced PAR, we employed emetine, a DNA replication inhibitor that prevents the formation of Okazaki fragments by uncoupling leading and lagging strand DNA replication [24,25]. Incubation with emetine completely blocked the appearance of hyperthermia-induced PAR (Figure 4A,B). This result did not reflect the non-specific effect of emetine on DNA replication because DNA replication inhibitors, such as aphidicolin and hydroxyurea, did not reduce the level of hyperthermia-dependent PAR (Figure 4A,B). This result also did not reflect the effect of emetine on PARP activity because emetine did not block PARylation at sites of DNA damage induced by hydrogen peroxide (Figure 4C).

Finally, we analyzed the existence of SSBs in S cells exposed to hyperthermia by in situ nick translation. In this assay, bacterial DNA polymerase I incorporates fluorescently labelled nucleoside triphosphates at sites of single-stranded DNA breaks or gaps. We found that short-term hyperthermia did induce a substantial number of SSBs in S-phase HEK293 cells (Figure 4D). Notably, this was in perfect agreement with our earlier studies that were performed with MCF7 cells [22]. Altogether, the data obtained confirmed our hypothesis and showed that hyperthermia-induced *c*-trapping of DNA replication proteins could inhibit maturation of Okazaki fragments, stabilize SSBs, and provoke a corresponding PARP-dependent DNA damage response.

## 4. Discussion

Hyperthermia has been used as an adjuvant treatment for radio- and chemotherapy for decades. Recent technological advancements, particularly in nanomaterial-based hyperthermia, have renewed interest in its use [1,2]. Aside from its effects on perfusion and oxygenation of cancer tissues [3], hyperthermia can enhance the efficacy of DNA-damaging treatments such as radiotherapy and chemotherapy [4]. Although it is believed that the adjuvant effects are based on hyperthermia-induced dysfunction of DNA repair systems, the mechanisms of this dysfunction remain elusive. A limited number of studies have demonstrated that hyperthermia can decrease the levels of some proteins involved in DNA repair [15,16,26,27]. Here, we attempt to propose a self-consistent and predictive explanation of how hyperthermia sensitizes cells to radio- or chemotherapy. We hypothesized and verified that hyperthermia induces non-functional trapping of particular proteins to chromatin, and thus exhausts their pool and alters molecular pathways that depend on these proteins. We identified an extensive list of factors that potentially undergo *c*-trapping in response to hyperthermia and verified some mechanistic predictions of the proteomics data obtained.

Our findings raise questions regarding the exact molecular mechanism of the trapping and its contribution to DNA repair or replication deficiency. In the context of hyperthermia, *c*-trapping can combine trapping of proteins to chromatin per se with the formation of protein aggregates strongly associated with chromatin. Since there is no clear experimental evidence for hyperthermia-induced DNA structural alterations such (e.g., B-to-Z DNA transitions, stabilization of G4 quadruplexes, or DNA cruciforms) in higher eukaryotes, it is more feasible to hold the view that the *c*-trapping represents the formation of protein aggregates strongly associated with chromatin. Such hyperthermia-induced aggregation has been discussed for a long time. Particularly, it was suggested that heat-induced protein unfolding could expose hydrophobic residues facilitating protein–protein interactions and co-aggregation of denatured and native proteins [28,29]. These protein aggregates were thought to associate with the nuclear matrix and nucleolus [28,30]. Nevertheless, the question of whether hyperthermia can alter DNA and/or chromatin structure deserves further investigation. Regardless of its nature, *c*-trapping leads to non-functional sequestration of proteins, and, in turn, to exhaustion of active protein pools. Consequently, this results in a deficiency of molecular pathways dependent on *c*-trapped factors. Of course, not all of the proteins identified in our proteomics analysis as *c*-trapped are exhausted or inactivated. This effect generally depends on the expression levels of particular proteins. The entire protein pool is not trapped, thus, low-expression factors are exhausted more rapidly and to a greater extent, making molecular pathways dependent on such low-level factors more sensitive to hyperthermia. This can partly explain why DNA repair and replication systems are among the most vulnerable to hyperthermia.

Based on proteomics data, we, for the first time, demonstrated that hyperthermia can inhibit Okazaki fragment maturation and, as a result, can stabilize SSBs and activate PARP-dependent DDR. The necessity of PARP for repairing these SSBs can also explain the combined lethality of hyperthermia and PARP inhibitors [26]. This example illustrates how the list of factors potentially exhausted in hyperthermia can help to develop new efficient combinatorial strategies for treating cancer.

## Supplementary Materials

The following are available online at https://www.mdpi.com/2073-4409/9/6/1423/s1, Figures S1 and S2: The full-length blots of Figure 1A and Figure 2A. Dataset S1: Proteins identified by LC-MS/MS analysis; Table S1: Top 20 significantly enriched Reactome pathway terms in the list of proteins depleted from the insoluble chromatin fraction upon hyperthermia; Table S2: Significantly enriched Reactome top-level (parent-level) pathway terms in the list of proteins undergoing *c*-trapping in response to hyperthermia; Table S3: Significantly enriched Reactome pathway terms related to DNA repair in the list of proteins undergoing *c*-trapping in response to hyperthermia.
