# Supplementary material for: Chromatin Trapping of Factors Involved in DNA Replication and Repair Underlies Heat-Induced Radio- and Chemosensitization
Source: Cells. 2020 Jun 8;9(6):1423. doi: 10.3390/cells9061423 (PMC7349668; doi:10.3390/cells9061423)
Supplement: Supplementary file 1 [file cells-09-01423-s001.zip › SupplementaryFigures.pdf]

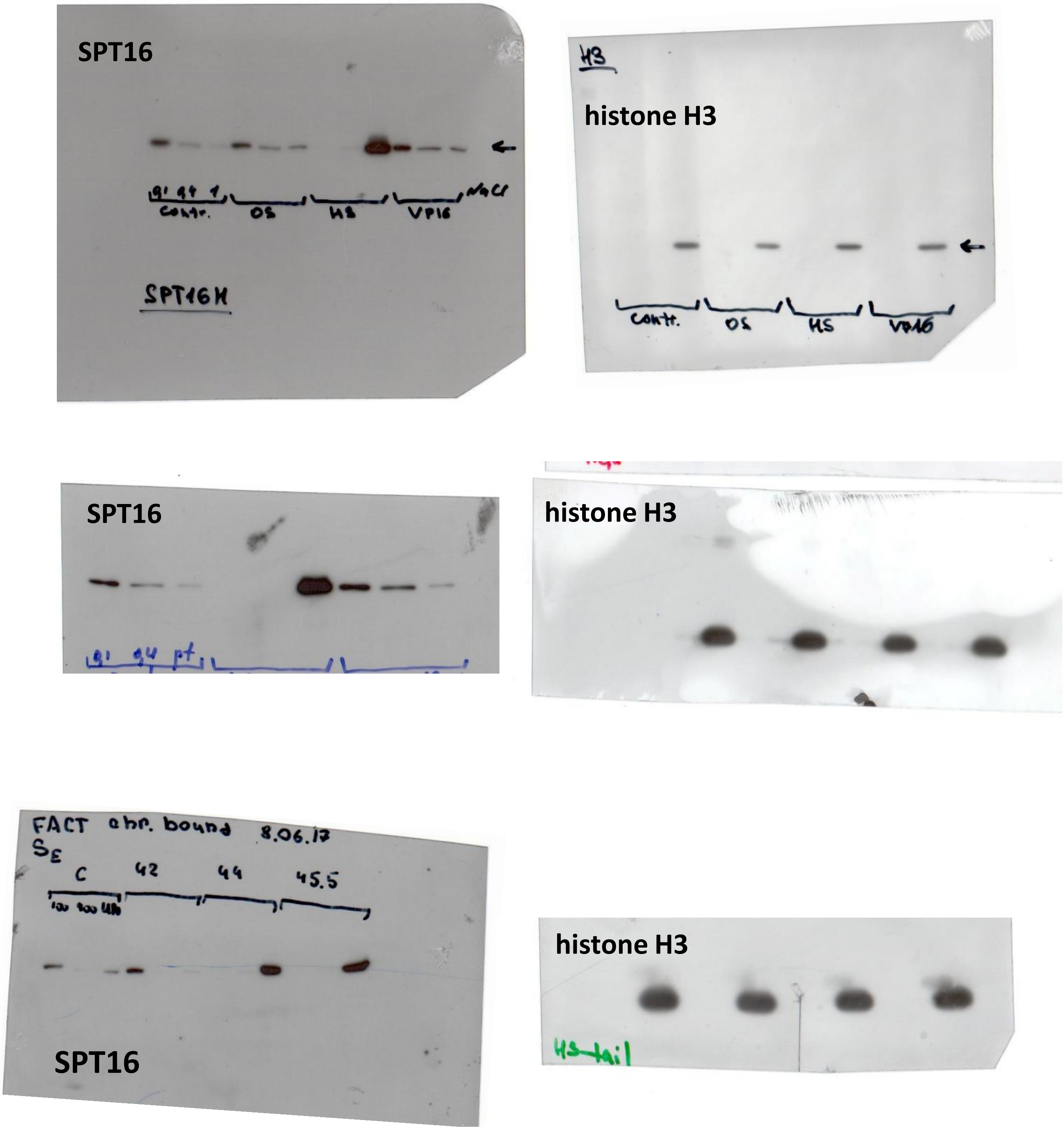

Figure S1. The full-length blots of Figure 1A. Western blot scanned films are shown.

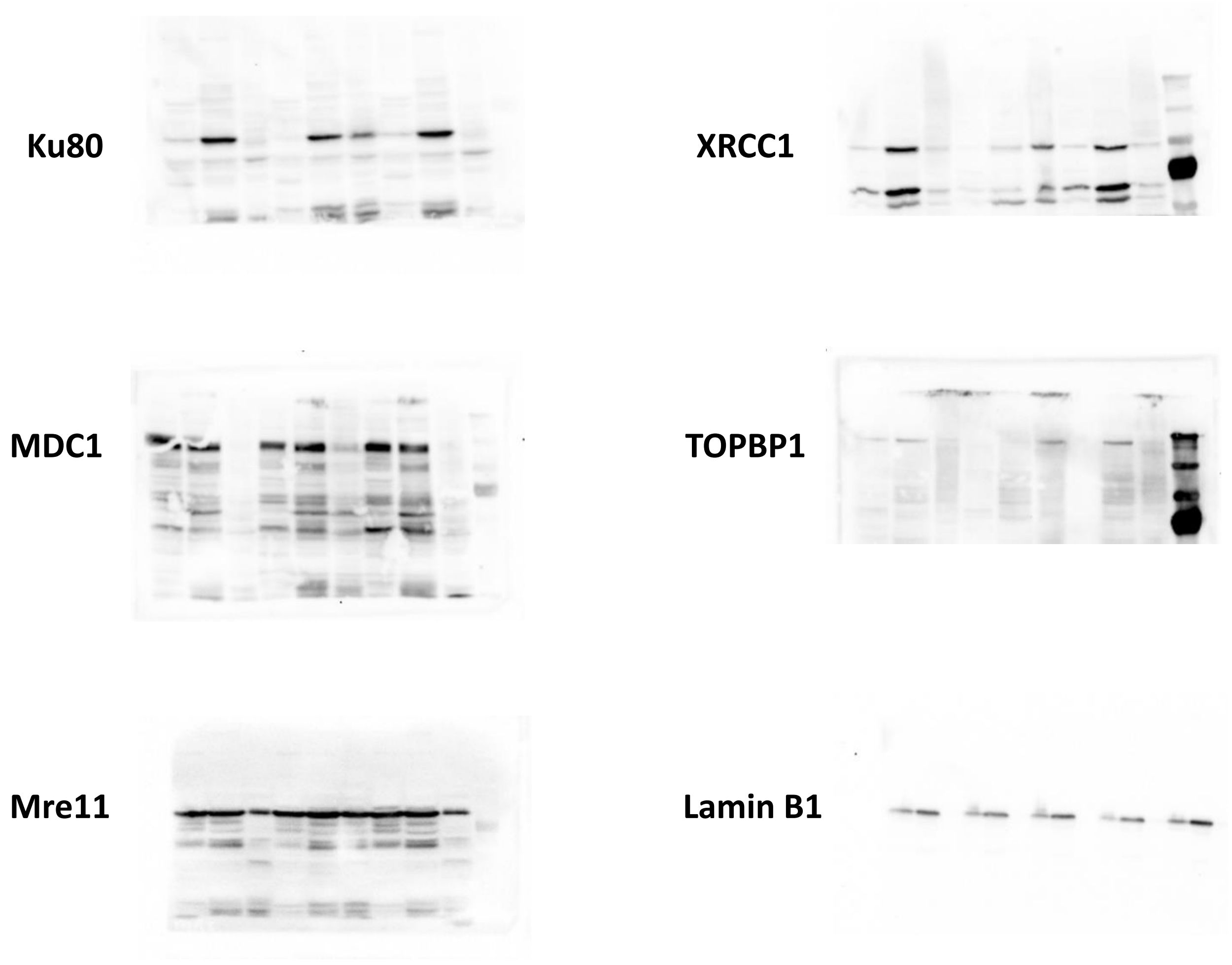

Figure S2. The full-length blots of Figure 2A. Images of the blots were obtained using iBright FL1500 Imaging System.
